# Expansion of opiate agonist treatment: an historical perspective

**DOI:** 10.1186/1477-7517-3-20

**Published:** 2006-07-21

**Authors:** Robert G Newman

**Affiliations:** 1 The Baron Edmond de Rothschild Chemical Dependency Institute of Beth Israel Medical Center, Albert Einstein College of Medicine of YeshivaUniversity, 555 West 57^th ^Street, 18^th ^Floor, New York, 10019, NY, USA

## Abstract

Untreated opiate addiction remains a major health care crisis in New York and in most other urban centers in America. Optimism for closing the gap between need and demand for treatment and its availability has greeted the recent approval of a new opiate medication for addiction, buprenorphine – which unlike methadone may be prescribed by independent, office-based practitioners. The likelihood of buprenorphine fulfilling its potential is assessed in the light of the massive expansion of methadone treatment more than 30 years earlier. It is concluded that the key, indispensable ingredient of success will be true commitment on the part of Government to provide care to all those who need it.

## 

Over thirty years ago an editorial appeared in *The New York Times *under the headline, "A Drug Success." [1] The focus was the New York City Health Department's Methadone Maintenance Treatment Program, which had expanded "so swiftly and so successfully...that there no longer are waiting lists for admission..." Recently, another medication – buprenorphine – was approved for treating opiate addiction, [2] and there is hope that it will allow many more patients to receive help. Critical to significant expansion of treatment capacity will be "clinician attitudes and the extent to which they embrace buprenorphine . . ." [3] However, to gauge the degree to which buprenorphine's potential will be realized it is important to consider the factors that went into the success of methadone in the early 70s.

New treatment services were being established throughout the country in those days, but the most dramatic increase took place in New York City, and it was due first and foremost to the vision and commitment of one man, Gordon Chase, the City's Health Services Administrator under then-Mayor John Lindsay. (Chase died in an auto accident in 1980 at the age of 47.) Chase, who had only a bachelor's degree and had never worked in the field of health care, was determined that every single heroin user would be offered prompt access to treatment; to achieve this goal he concluded that methadone maintenance would have to be the cornerstone of the City's efforts. He acknowledged readily that he knew very little about methadone (few at the time knew more – methadone had been introduced just five years before [4]); he had been persuaded, however, that without methadone the vast majority of those who needed and were willing to accept help would be abandoned.

Chase peremptorily dismissed the litany of reasons staff gave in urging him to "go slow": rapid expansion of methadone treatment had never been attempted, and could be a widely publicized disaster that would undermine treatment efforts everywhere; individualization of care and "comprehensive ancillary services" were considered indispensable components of treatment and required extensive time and staff training to establish; etc. Indeed, virtually every experienced professional in the country rejected the very concept of expansion on a massive scale. This may explain why Chase asked the author to implement his vision of "treatment on request" for all addicts; I was a resident in Public Health at the time, whose only prior medical training had consisted of two years of general surgery, and whose administrative background was limited to a few months directing the New York City component of a national nutrition survey.

The consistent response by Chase to the nay-sayers within and outside City Government was to ask: "How convincing will your concerns and criticisms be to parents whose children sought help but were turned away, and subsequently died of an overdose?" His argument was compelling,. In any event, Chase prevailed and within two years the City Health Department had established a program with an active enrollment of approximately 11,000! Concomitantly, the Health Department spurred ("shamed" is probably a more accurate term) other methadone and drug-free providers in New York and elsewhere to increase their own capacity markedly.

The net result for the City of New York was dramatic: a sharp reduction in addiction-related property crime, drug arrests, hepatitis and deaths attributed to drug dependence. [5] As for the Health Department's new methadone program in particular, whether measured by retention rates, employment, drug use, health status or any other parameter, the extensively documented outcomes were every bit as good as those of other addiction treatment services. [6]

The New York City Health Department program was financed entirely by City and State funds and by Medicaid reimbursement for eligible patients. The Federal Government provided neither fiscal nor moral support for the City's unprecedented response to opiate addiction, the most important clinical and public health challenge of the day. As for the rest of the country, without the demonstration by New York that massive, rapid expansion was feasible, it is likely that things would have proceeded at a snail's pace.

Tragically, since the mid-70s there has been little if any further increase in addiction treatment capacity of any kind in America. Roughly the same 20 percent of the estimated heroin addicted population receives care today as did then – before the onset of AIDS. [7] Undoubtedly the greatest obstacle to accommodating more patients has been the absolute monopoly on methadone maintenance that has been given to "programs;" independent, office-based practitioners are excluded from the field by law – a restriction on prescribing that applies to no other medication in the US pharmacopoeia.

## A "new" treatment option

*The New York Times *recently reported [8] that an estimated 36,000 patients receive methadone in the City – essentially the same number as three decades ago (according to one source, 34,000 patients were being treated with methadone maintenance in 1974 [9]). At the same time, *The Times *noted optimistically that prescriptions for buprenorphine are "expected to soar in the coming years" (one year earlier the paper had run another optimistic story on buprenorphine under the headline, "New drug promises shift in treatment for heroin addicts" [10]). In fact, however, if the past is prologue, the acceptance and utilization of buprenorphine may be a long time coming. As early as 1978 it had been described as a medication with "a unique pharmacology with immediately obvious therapeutic application as a maintenance drug in narcotic addiction" [11]. And yet, to make this "immediately obvious" medication a reality " . . . took considerable financial commitment from NIDA [National Institute on Drug Abuse], more than two decades of dedicated effort by myriad researchers and practitioners, and the collaboration of a willing and savvy pharmaceutical manufacturer. It also literally took an act of congress" [3].

The breakthrough itself, when it finally came, was not pharmacological but regulatory. Unlike methadone, buprenorphine could henceforth be prescribed for opiate dependence by any physician who is "certified." Certification requires nothing more than an application and demonstration that the physician has completed an eight-hour training course (which also is available on-line). While the demands imposed on "methadone programs" are undiminished, and office-based physicians continue to be barred from making methadone available to their patients, buprenorphine can be prescribed to a new patient for a full month, and in some states (e.g., New York) the prescription can be refillable without further physician-patient contact for five additional months. Surely not good medical practice – but in contrast to methadone, not illegal! The one restriction that makes treatment with buprenorphine exceptional is that no physician or group practice may treat more than 30 patients at a time (this limit, as it applies to group practices, was eliminated in a bill passed by Congress and signed by the President in August, 2005 [12].

Despite all the hype, the ease of certification and the relative absence of regulatory constraints, there's little to cheer about. Notwithstanding the very considerable effort of the Federal Government, and the extensive advertising and public relations campaign of buprenorphine's manufacturer, the percent of previously untreated opiate-dependent individuals that receives this medication appears to be miniscule. Worse, it's by no means clear that anyone cares; no Federal targets have been announced, and no one seems to be measuring the increment in patient numbers (and if they are, they are not talking, which also bodes ill). Of course, it is likely that the manufacturer is following its sales very, very closely, but it too has released no data.

One major barrier to significant expansion of addiction treatment with buprenorphine is the persistent mixed message sent by Government. We can hardly expect physicians, patients or the public at large to embrace treatment with one medication (buprenorphine), when Government itself continues to reflect and reinforce the stigma towards treatment with another medication (methadone) for the same patients and the same disease. We'll never see significant numbers of physicians – i.e., "mainstream medicine" – prescribe buprenorphine when methadone must, by law, be associated with a fully panoply of "comprehensive ancillary services," frequent urinalysis, stringent restrictions on "take-home privileges," and inspection and approval of all providers by "accreditation agencies." In addition, of course, there continues to be an absolute bar, regardless of circumstances, against treatment with methadone by independent office-based practitioners.

The experience three years after buprenorphine was approved speaks for itself. Less than 500 prescriptions for buprenorphine, from all sources, were written in New York City during the month of June, 2005. [13] If each prescription were for an unduplicated individual, the total recipients of this medication would be one-quarter of one percent of the estimated 200,000 untreated heroin-dependent population of the city. [14]

## Conclusion

We need Government to give strong, unqualified support to the premise that addiction is a chronic medical condition. It must acknowledge forthrightly that neither buprenorphine nor methadone nor any other treatment modality, medication-based or drug-free, is a "cure." At the same time, it must stress the fact that addiction is eminently treatable. (The same reality of "treatable but not curable" applies to all chronic illnesses.) Above all, however, we need leaders with the commitment, pragmatism and common sense that Gordon Chase personified. Sadly, such traits are rarely evidenced today by those who influence and implement policy – in government, academia or the private sector. Meanwhile, hundreds of thousands of opiate dependent people in New York and throughout the country continue to suffer and die, and society at large bears the associated fiscal and human costs. It is high time to reconsider the rhetorical question Chase posed almost 35 years ago: Are our rationalizations for tolerating the *status quo *truly persuasive? Would they be accepted by those who are suffering and dying as a consequence of inaction?
